# The Evolution of Masturbation in Birds

**DOI:** 10.1002/ece3.73693

**Published:** 2026-05-31

**Authors:** Chloe Heys, Kevin Arbuckle, Matilda Brindle, Tom A. R. Price

**Affiliations:** ^1^ School of Pharmacy and Biomedical Sciences University of Lancashire Preston UK; ^2^ Institute of Infection, Veterinary and Ecological Sciences, Crown Street University of Liverpool Liverpool UK; ^3^ Department of Biosciences, Faculty of Science and Engineering Swansea University Swansea UK; ^4^ Department of Biology, Life and Mind Building University of Oxford Oxford UK

**Keywords:** adaptive evolution, masturbation, sexual behaviours, sexual outlet

## Abstract

Most theories on sexual behaviours are based on adaptive explanations. However, masturbation, a common but scarcely discussed sexual behaviour, appears a Darwinian puzzle. Why would individuals waste valuable resources such as time, energy and, for males, sperm? We combined targeted surveys with information from published accounts to test hypotheses about why masturbation occurs, using a phylogenetically broad dataset on the presence or absence of masturbation across 120 bird species. We find masturbation is widespread in birds, but strongly phylogenetically conserved, typically being fixed (as present or absent) or nearly so within broad clades. Masturbation is more common in males, though the widespread evidence for masturbation in females suggests that maintaining fresh sperm in testes cannot be the single explanation. We find no difference in masturbation occurrence between juveniles and adults, further suggesting that it does not solely represent practice copulations before maturity. Species with indiscriminate matings are more likely to masturbate than socially monogamous species or species with long‐term pair bonds. Importantly, as masturbation is more commonly reported in wild than captive birds, we find it is therefore not a negative or maladaptive response to captivity. Instead, it is part of a wider repertoire of sexual behaviours exhibited in birds. Overall, our data are consistent with masturbation serving as an outlet for increased sexual arousal, a means of increasing reproductive success through postcopulatory selection, or both.

## Graphical abstract

Masturbation has been sporadically documented across animal groups, yet the evolutionary origins and persistence of this sexual behaviour are largely unexplored. Through combining targeted surveys with published accounts, we test hypotheses about why masturbation occurs using a phylogenetically broad dataset on the presence or absence of masturbation across 120 bird species. We find masturbation is widespread in birds and suggest it serves as a sexual outlet, a means of increasing reproductive success, or both.

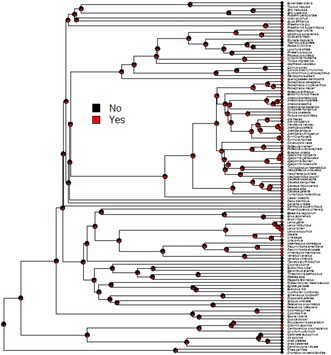

## 1 Background

Non‐reproductive sexual behaviours in animals can widely vary in complexity and function. The ability to determine whether they serve an adaptive function and how they persist across evolutionary time is vital to enhance our understanding of these behaviours. Masturbation, also described as autosexual behaviour, is a primary example of a non‐reproductive sexual behaviour which has been sporadically documented in non‐human animals (which we will hereon refer to as animals), yet the adaptive function of this behaviour and its prevalence across the animal kingdom is still poorly understood.

Aside from recent interest (Roth et al. 2023; Brindle et al. 2023), masturbation in animals, which we define as sexual stimulation of the genitals by self‐manipulation, has received relatively little attention in scientific research. There could be several reasons for this apparent gap in the scientific literature surrounding this behaviour: its consideration as a taboo subject, the assumption that masturbation is a behaviour limited to humans, or at most, primates, and issues with defining masturbation behaviour. For example, it has been previously defined as ‘manual stimulation of the sex organs to ejaculation’ (Waterman 2010), which unhelpfully excludes females. It is therefore inadequate to use as a blanket definition. Masturbation has been sporadically documented in an array of male and female vertebrates, including Cape ground squirrels ( *Xerus inauris* ; Waterman 2010), equids (McDonnell et al. 1991), bottlenose dolphins (*Tursiops* sp.; Linden 2011), marine iguanas (Wikelski and Bäurle 1997) and Adelie penguins ( *Pygoscelis adeliae* ; Russell et al. 2012), but reports are highly concentrated within primates, such as Japanese macaques (*Macaca fuscata yakui*; Thomsen and Soltis 2004), gibbons (*Hylobates*; Mootnick and Baker 1994), Phayre's langur ( *Trachypithecus phayrei* ; Shalauddin et al. 2021) and chimpanzees (Havercamp et al. 2022). Despite masturbation seemingly occurring regularly across a range of vertebrate taxa, little is known of the evolutionary origins of this behaviour outside of the Primates.

Animals exhibit several non‐reproductive sexual behaviours, such as sex during pregnancy (Clarke et al. 2022) or between same‐sex individuals, with the latter widely reported in birds (Bagemihl 1999). Both proximate and ultimate explanations for these behaviours are still widely debated. Masturbation, another example of a non‐reproductive sexual behaviour, similarly falls into this evolutionary quandary. Why would individuals choose to masturbate and spend valuable resources such as time, energy, and in the case of males, sperm, over mating with a viable partner?

With instances of masturbation increasingly documented across the animal kingdom, several theories have aimed to explain the occurrence and persistence of this behaviour. Some hypotheses, such as the Sexual Outlet Hypothesis, suggest that masturbation is not adaptive but is simply a by‐product of selection for the neuroendocrine mechanisms that lead to increased sexual arousal, particularly for organisms with multiple mates (also known as promiscuous mating systems; Dixson and Anderson 2004). However, this proximate mechanism for masturbation may not be mutually exclusive with adaptive explanations.

The Postcopulatory Selection Hypothesis, an umbrella term used to provide an adaptive explanation for masturbation, suggests that masturbation serves to increase reproductive success in a variety of ways. For example, masturbation can increase sexual arousal and/or orgasm, which can in turn facilitate cryptic female choice. Sexual arousal is shown to increase vaginal pH to create a more hospitable environment for sperm in primates (Meston 2000), and the uterine contractions associated with orgasm also aid the passage of sperm (Puts and Dawood 2006; Suarez and Pacey 2006). These mechanisms may therefore allow females to use pre‐ or post‐copulatory masturbation to increase reproductive success by a given male (Brindle et al. 2023). In males, masturbation can aid sperm replenishment—removing and replacing old sperm to increase the likelihood of successful fertilisation in subsequent matings with conspecifics (Brindle et al. 2023). In both sexes, masturbation could serve as a communication mechanism for advertising to a potential mate, warning rivals (Waterman 2010), or reinforcing pair bonds (this would be of particular importance for monogamous species). In each of these cases, masturbation would prove adaptive.

Similarly, for species that exhibit multiple matings, the adaptive function of masturbation as removal of old sperm would be of importance, so that when individuals have the opportunity to mate with a potential partner, the sperm transferred is replenished and of higher quality, which would therefore be more likely to result in successful fertilisation (Thomsen and Soltis 2004). However, this would not explain female masturbation. Aside from mating system, the timing of masturbation is also closely correlated to understanding its adaptive function. For example, there has been some evidence to support the theory that masturbation serves as a pathogen avoidance mechanism. First explored in Cape ground squirrels, masturbation was found to occur post‐copulation in this highly promiscuous species (Waterman 2010) as a form of genital grooming by cleansing the reproductive tract with ejaculate. This has recently been explored in primates, with males from multi‐male multi‐female mating systems found to masturbate to reduce the risk of STIs (Brindle et al. 2023). The Pathogen Avoidance Hypothesis therefore requires masturbation to occur only post‐copulation. Where masturbation occurs outside of these times, it could instead be argued that masturbation is a flexible behaviour, with different functions according to life history stage. For example, juveniles may masturbate to practice copulation in order to increase fertilisation success when they achieve maturation, as has been documented in spectacled parrotlets ( *Forpus conspicillatus* ; Garnetzke‐Stollman and Franck 1991).

Masturbation is therefore undeniably present in animals, but is it clustered in species with shared behavioural phenotypes? It could be argued that occurrence and prevalence of masturbation is linked to the mating system of an organism. Whilst social monogamy and biparental care are the dominant mating systems of birds, they are well known to exhibit complex mating behaviours to attract a potential mate (Johnson and Burley 1998). Despite this diversity of mating behaviours, the role of masturbation in sexual selection in birds has not been examined in scientific literature, with brief reports limited to historical accounts in Adelie penguins from an Antarctic explorer (Russell et al. 2012), spectacled parrotlets ( *Forpus conspicillatus* ; Garnetzke‐Stollman and Franck 1991) and few others. Yet despite this, masturbation in birds has been widely anecdotally reported amongst hobbyist keepers, breeders and enthusiasts. For example, there are numerous online forums that are dedicated to understanding masturbation in pet birds (ParrotForums 2016).

Masturbation in birds typically occurs through an individual rubbing their cloaca against an object such as a twig or toy (Garnetzke‐Stollman and Franck 1991) and is often accompanied by vocalisations or wing flapping. The cloaca is the orifice that is used for three functions—defecation, urination and reproduction. In males, the cloaca is used to transfer sperm to the female, whereas in females, the cloaca serves to accept sperm and lay eggs (Bellairs and Osmond 2014). One potential reason for the lack of scientific studies examining masturbation in birds could be because the cloaca is thought to have lower sensitivity through reduced nerve clusters (Palmieri et al. 2003), therefore suggesting that birds may not achieve orgasm in the same way that mammalian species do. Indeed, the phallus (penis or clitoris) is thought to have evolved from a single origin in amniotes, from an anlage on the ventral side of the cloaca (Gredler et al. 2015). Therefore, the idea that birds may have sex for pleasure has likely been dismissed prematurely, and as this appears to be a proximal motivation for masturbation in humans, this may partially explain the lack of research on masturbation in birds.

Perhaps the most dominant theory is that masturbation has evolved as a maladaptive response to captivity in birds. As bird keeping and/or breeding is a well‐known and respected hobby across the world, care of these captive birds is of the utmost importance. The evolutionary causes and persistence of masturbation have been scarcely examined by scientists, yet the anecdotal literature of masturbation occurring in captive birds is vast. As a result, masturbation is often described as a ‘problem’ behaviour in birds. Several avian surgeons have reported masturbation occurring in tandem with aggressive or harmful behaviours such as biting or screaming, and even self‐mutilation and feather plucking (Rosen 2012). Frequent masturbatory behaviour has also arguably been linked to cloacal disease in birds (Rosen 2012). Therefore, as part of this maladaptive response to captivity, masturbation could serve as a stress‐reduction mechanism and/or a response to sexual frustration, as birds are often housed without a potential mate. Preventing or ceasing masturbation is therefore a focal topic of concern for avian veterinary professionals, in order to prevent these damaging behaviours and improve the welfare of captive birds. The idea that masturbation is a problem behaviour that needs treatment has become engrained as folklore husbandry within the avian community—a form of husbandry/practice that is based on common knowledge or experience, rather than evidence (Arbuckle 2013).

In the current study, we investigate the prevalence and persistence of masturbation across avian taxa. Using a combined approach of a comprehensive literature review, coupled with targeted questionnaires and a citizen science approach, we gathered reports of whether masturbation occurs in a range of species across taxonomic orders. Presence or absence of this behaviour was recorded alongside several other variables such as age, sex, origin, social environment and physical condition. Our findings are examined in conjunction with both leading hypotheses and our own suggestions, with the aim to elucidate the ultimate mechanism for phylogenetic occurrences of this behaviour in birds and provide further evidence of the ubiquitous occurrence of masturbation within the animal kingdom.

## 2 Methods

The few studies examining masturbation in animals have used an array of definitions to describe masturbation, and these are often geared towards the study organism. We define masturbation as sexual stimulation of the genitals by self‐manipulation. Since we explicitly define masturbation as sexual stimulation, behaviours such as cleaning, preening or scratching—all of which are easily differentiated via observation—are therefore excluded. Our definition includes individuals using inanimate object(s) as well as the birds' own limbs, and does not necessitate masturbation to ejaculation in males. It is important to note, however, that there are no occurrences of own‐limb stimulation within our dataset that may complicate assessment of, for instance, scratching vs. masturbation. Methodology was conducted in accordance with relevant guidelines and regulations and approved by the University of Liverpool and the British and Irish Association of Zoos and Aquariums (BIAZA) ethical committees.

Reports of masturbation in birds are most commonly recorded from non‐experts using a variety of platforms. As such, we used several approaches to determine how widespread instances of masturbation were documented across avian taxa. These were: (i) literature review, (ii) surveying avian experts, and (iii) online reports and communities. A database was then created that collated information detailing species, age (adult/juvenile), sex (male/female), origin (wild/captive), species mating system (including whether species were socially monogamous, lekking, had multiple mates, or for monogamous species, had long‐term pair bonds), social environment (mixed species/solitary/opposite sex/same sex housing) and physical condition (good/bad). For captive species we also recorded the rearing origin which we classified as either parent or hand reared (using human intervention). We defined ‘long‐term pair bond’ as species which pair with the same individual across multiple mating seasons. Social environment was used to record if individuals were housed with others of the same/different sex and the same/different species, and how many were in the group housing situation. Mating system information was gathered from the scientific literature and referenced accordingly. Informed consent for use in this study was obtained from all contributing participants (experts and reports gathered from individuals from online communities).

### 2.1 Literature Review

We first performed a literature review to compile published and peer‐reviewed accounts of masturbation across avian taxa. We performed electronic searches of the literature in line with the methodology from (Mengist et al. 2020). The search terms used were: ‘masturbation’, ‘solo‐sex’, ‘bird’, ‘mating + behaviour’, ‘mating + system’. After identifying articles of interest using our search terms, all articles within the final library were read in full and included in the data collection if they met the final criteria and documented or discussed instances of masturbation in avian taxa (if masturbation occurred or explicitly was not observed in a given species).

### 2.2 Avian Experts

We produced a questionnaire as a tool to survey avian experts in order to quantify how widespread masturbation is across avian taxa. The questionnaire disseminated is displayed in the Data S1. We surveyed experts from academia who either currently or previously conduct research in birds, as well as other professionals and practitioners such as zookeepers. In association with the British and Irish Association of Zoos and Aquariums (BIAZA), this questionnaire was disseminated to all zoos registered within this organisation. This approach was selected as bird keepers have extensive experience of working in close quarters with their study organisms and could therefore confidently state whether or not they had observed masturbation in their study species. As such, we requested data only for species that the respondent felt they had substantial experience and time observing and could therefore confidently distinguish masturbation from other behaviours. Masturbation was classed as present in species for which a single confirmation of masturbation was recorded. A species was classified as non‐masturbatory if the respondent had never observed this behaviour before and there were no submitted responses to say the contrary.

### 2.3 Online Reports and Communities

There is a stark disparity between the number of published accounts reporting masturbation in avian species (whether wild or captive), in comparison to its widespread documentation in unpublished formats from bird keepers, breeders and enthusiasts, and within online communities. As such, a vital component of this research was to integrate the extensive knowledge from these individuals and groups with published accounts, to determine how widespread this behaviour is observed across avian taxa. We therefore used a citizen science approach to survey online communities of bird keepers, breeders and enthusiasts for evidence of this behaviour, using the same questionnaire that was sent to avian experts. Further, we extended this methodology to conduct a comprehensive review of documented accounts of masturbation in avian taxa across online communities. We reviewed forums, social media sites (Facebook) and the online video sharing platform, Youtube. For all online data collection accounts, we had set inclusion criteria that posts had to reach in order to be included in the data collection. The minimum requirements were species, sex (either clearly stated or apparent through photographic evidence, such as in sexually dimorphic species), and an accurate written, photographic or video description of the individual(s) masturbating. Any posts that did not reach these minimum requirements were not included in the data collection or any subsequent analysis.

### 2.4 Phylogenetic Comparative Analysis

All analyses were conducted in R 3.4.1 (R Core Team 2021) and basic handling and manipulation of phylogenies was carried out using the packages ape 4.1 (Paradis and Schliep 2019), phytools 0.6.20 (Revell 2024), and functions incorporated into windex 2.0 (Arbuckle and Minter 2014). We obtained a sample of 500 phylogenetic trees of birds from https://birdtree.org/ (Jetz et al. 2012) with 250 each based on the (Hackett et al. 2008; Ericson et al. 2006) backbones to enable us to account for substantial phylogenetic uncertainty in the higher order relations of birds. For analyses requiring a single phylogeny rather than a subset we calculated a maximum clade credibility (MCC) tree using phangorn 2.2.0 (Schliep 2011).

We first assessed the suitability of our key trait (presence vs. absence of masturbation) for modelling in a phylogenetic comparative context by calculating the phylogenetic imbalance ratio (PIR; Gardner and Organ 2021) using windex 2.0.8 (Arbuckle and Minter 2014). PIR values range between 0 and 1, with lower values indicating greater suitability of the data for model fitting. Although advising against strict thresholds, Gardner and Organ (2021) suggested that PIR < 0.1 is likely to indicate that parameter estimation is feasible from comparative models. We calculated PIR for the presence vs. absence of masturbation in a species from our dataset, and the resulting values strongly suggest that our data are adequate for modelling using phylogenetic comparative methods (PIR = 0.003).

We estimated phylogenetic signal as the proportion of residual variance explained by phylogeny in a phylogenetic mixed model (pGLMM) with presence/absence of masturbation as a binary response variable (i.e., with family = ‘categorical’), no explanatory variables, and the phylogeny as a random effect. This approach allowed us to make full use of the data by allowing estimation of phylogenetic signal accounting for intraspecific variation, unlike many other measures which can typically only handle a single datapoint for species in their implementation. We fit this pGLMM in a Bayesian framework using MCMCglmm 2.24 (Hadfield 2010), setting priors on all parameters to be inverse Wishart distributions with V = 1 and nu = 0.002. The MCMC was initially run for 11,000,000 iterations, the first 1,000,000 of which were removed as burn‐in, and the remainder were sampled every 10,000 iterations to give a posterior distribution of 1000 samples The effective sample sizes for every parameter were checked and if any parameter estimate had ESS (Evolutionarily Stable Strategy) < 200 we increased length, burn‐in, and sampling interval by a factor of 10 to achieve reasonable effective sample sizes while keeping the total number of posterior samples at 1000. We also examined trace plots of likelihood and parameters, autocorrelation plots, and Geweke plots to ensure there were no patterns in the chains that would suggest poor exploration of the parameter space. All parameters passed Heidelberger and Welch's convergence diagnostic test, and all autocorrelation values were < 0.1 at a lag of 10 except for the pGLMM for cooperative breeding. We calculated the proportion of residual variance attributed to phylogeny using the posterior mean estimates of the phylogenetic component divided by the sum of this and the residual error component.

We estimated ancestral states for masturbation using Bayesian stochastic mapping (Bollback 2016; Huelsenbeck et al. 2003) as implemented in phytools, which allows incorporation of both intraspecific variation and phylogenetic uncertainty via providing a set of trees. We fit an ‘all rates different’ model based on 5000 simulations (10 on each phylogeny in our posterior sample), with the root node being sampled from the conditional scaled likelihood distribution at the root. We extracted the numbers of gains and losses and the transition rates of the estimated model from the output, and used a Wilcoxon matched‐pairs test on the posterior distributions to compare the number and rate of gains vs. losses of masturbation over evolutionary time. A summary of model outputs is reported in the Data S2.

To examine predictors of masturbation in birds we fit a series of pGLMMs as described above in the context of estimating phylogenetic signal but including explanatory variables representing our hypotheses. Because of the large number of parameters needed to fit all explanatory variables in a single model, which prevented adequate parameter estimation, we ran each model with a single explanatory variable. The latter were: age (adult/juvenile), sex (male/female), origin (wild/captive), species mating system (characterised in four models, with presence or absence of social monogamy, long‐term pair bonds, lekking, or multiple mates), social environment (mixed species/solitary/opposite sex/same sex housing), condition (good/bad), and rearing condition (parent/hand‐reared) for data in captivity. Please note, we use the term ‘multiples mates’ to encompass any multiple mating system, such as polygynous (one male, multiple females), polyandry (one female, multiple males) or polygynandry (multiple males and females), including when individuals mate many times with the same, or different individuals. We were unable to include two of our planned models. The first of these, with presence/absence of cooperative breeding as an explanatory variable, failed to perform adequately according to our diagnostic checks. The second, investigating an effect of condition (poor/good), was unable to be run at all as there were no records of poor condition birds in our dataset, but this at least demonstrates that birds in good condition do masturbate and it is not exclusively associated with birds in poor condition. We present estimates of *p*‐values as pMCMC from our MCMCglmm models, which is defined as twice the posterior probability that the estimate is negative or positive (whichever probability is smallest) (Hadfield et al. 2013). We used pMCMC to inform our assessment of statistical significance when interpreting our results.

## 3 Results

We recorded data for a total of 120 species across 22 avian orders. We compiled over 200 records from the literature, questionnaire responses and personal communications from a range of ornithologists, avian experts, breeders and keepers to produce the largest dataset of masturbation in birds to date. A summary of sample sizes is reported in Data S3.

**FIGURE 1:**
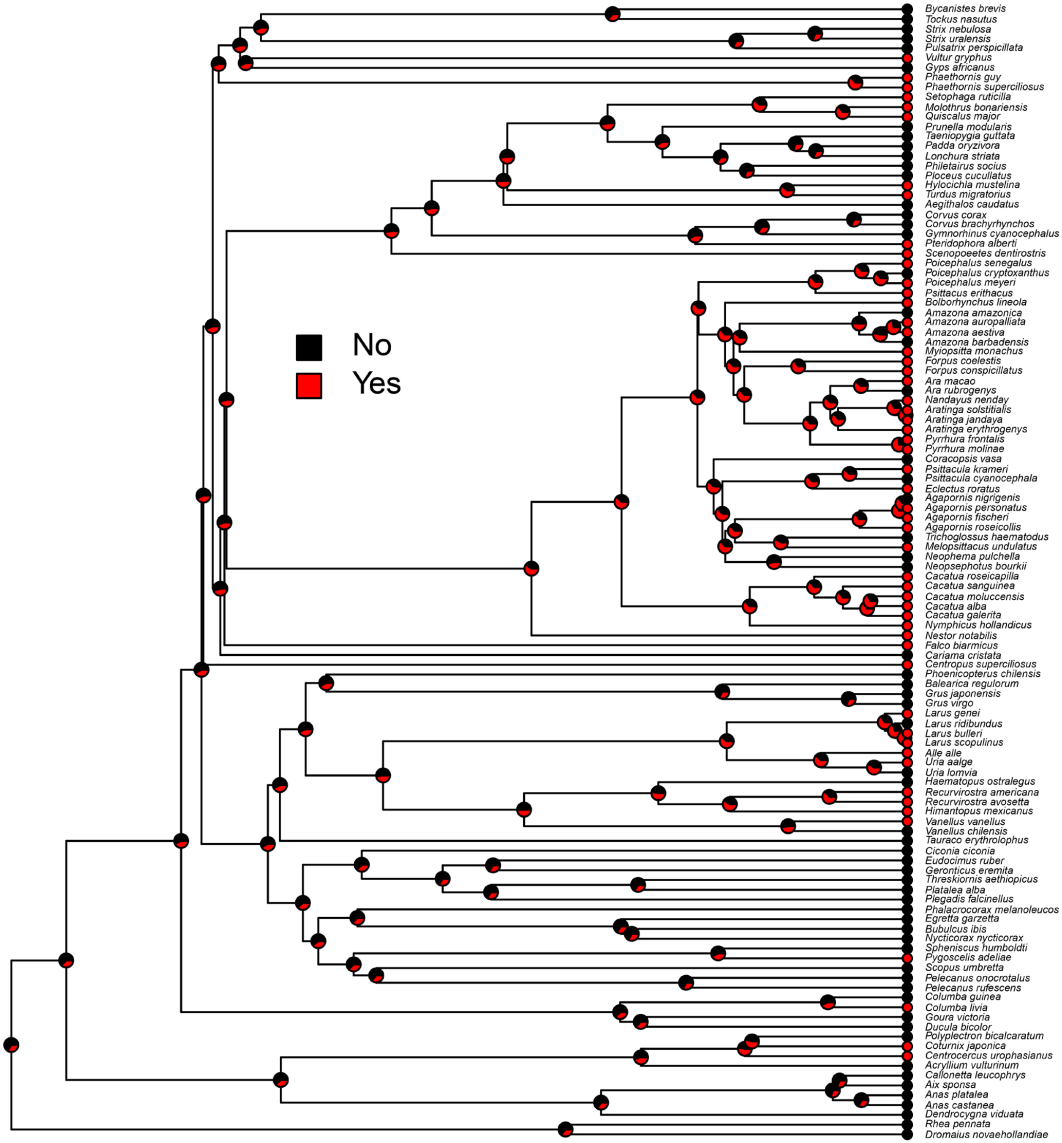
Ancestral state estimation showing the estimated probability of masturbating (red) or not (black) over one of the phylogenetic trees included in our analyses. Circles at the tips show the data rather than estimates; note the substantial phylogenetic clustering of masturbation behaviour.

**FIGURE 2:**
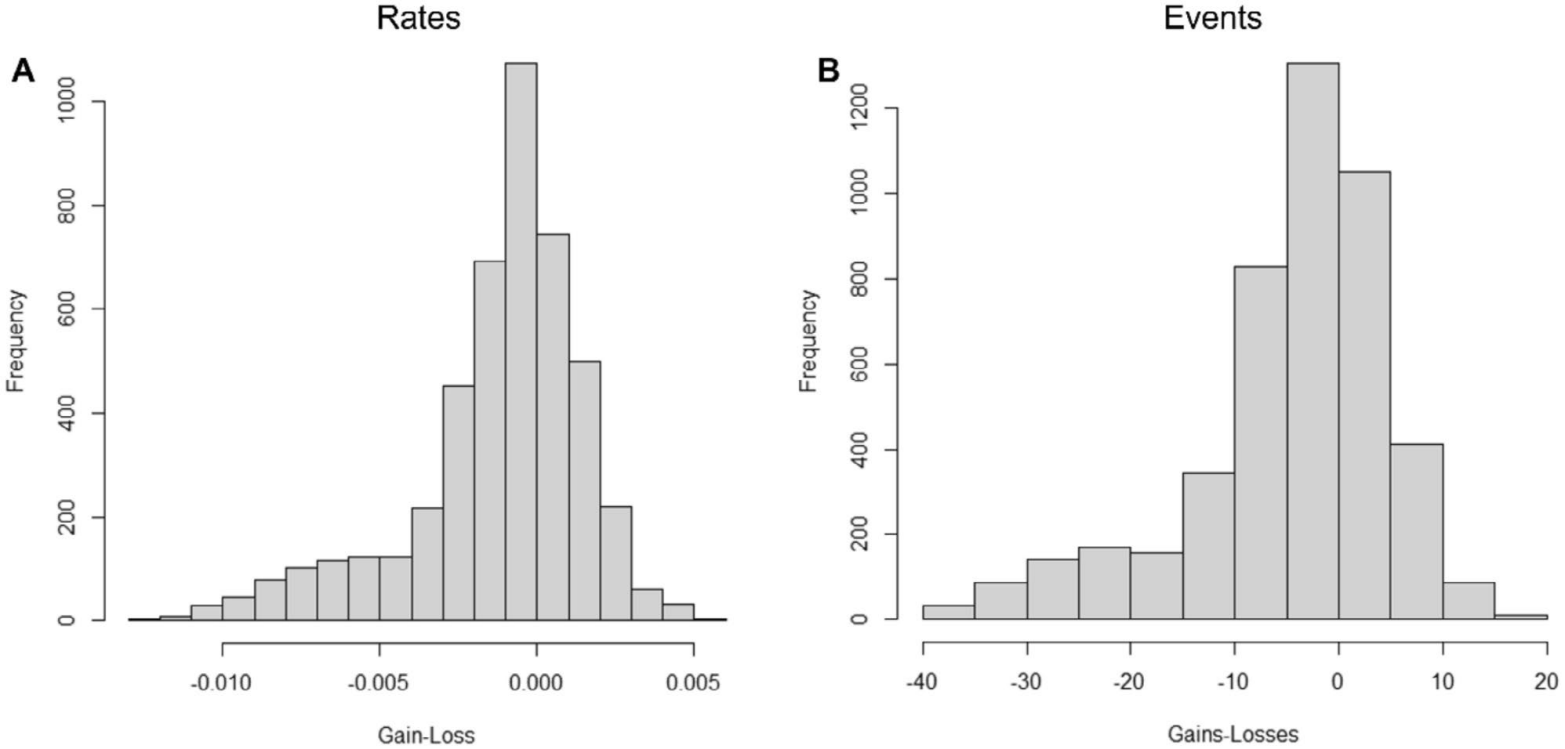
Posterior distributions of the difference between gains and losses of masturbation behaviour, including differences in transition rates (A) and the number of events (B). Positive values indicate higher rates of gain than loss (A) or more gains than losses (B), whereas negative values suggest the opposite. Note that our estimates support a tendency for masturbation behaviour to be lost rather than gained, but this could be influenced by unobserved masturbation in some species (see text).

The occurrence of masturbation is widespread across avian taxa and is strongly phylogenetically conserved (phylogenetic signal = 0.998), suggesting that we are indeed picking up a meaningful evolutionary signal as the distribution of masturbation is phylogenetically structured (not random) (Figure 1). We find a higher rate of evolutionary losses than gains (Wilcoxon matched‐pairs test: *p* < 0.0001; Figure 2) with masturbation typically characterising broad clades. Our results also suggest that masturbation was most likely to be absent from the common ancestor of birds (P_masturbation_ = 0.34 cf. P_no masturbation_ = 0.66), but this is only approximately twice as likely as the converse which leaves substantial uncertainty. We recorded the highest number of responses from the order Psittaciformes (parrots) but our data included wide representation across phylogenetic orders.

**FIGURE 3:**
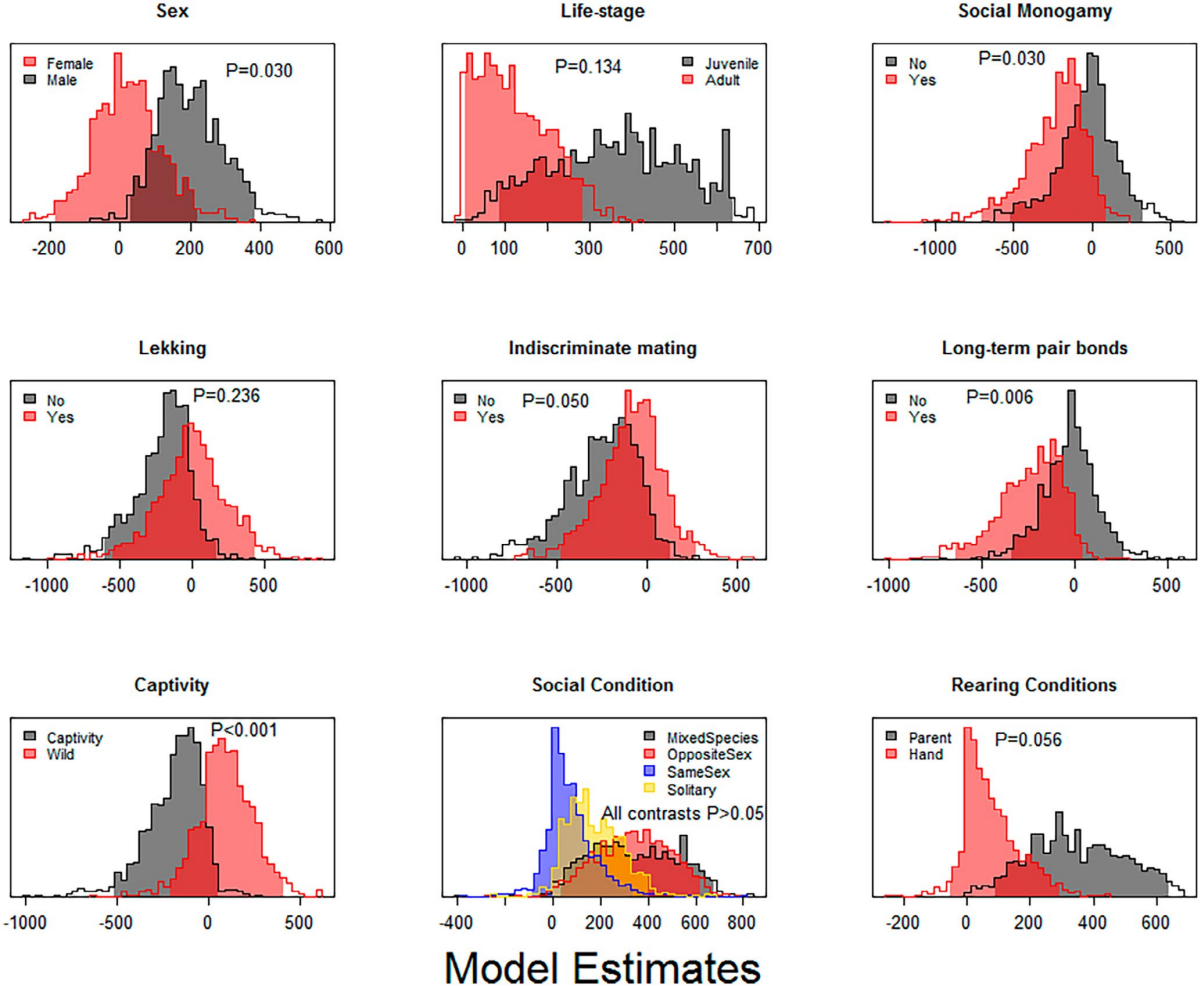
Posterior distributions of estimated coefficients from MCMCglmm models predicting the presence of masturbation by each of nine explanatory variables. P refers to pMCMC values from the model, but note that these are estimated from 1000 posterior samples so exact pMCMC values are not given for pMCMC < 0.001 as this is the limit of precision.

We documented numerous reports of masturbation occurring in both males and females within all orders we gathered data for. However, our results indicate that males are statistically significantly more likely to masturbate than females (55% of male records involved masturbation compared to 36% of female records; β = 177.63, 95% HPD (Highest Posterior Density) interval = [29.38, 356.57], pMCMC = 0.030; Figure 3). No statistically significant differences were observed in age class (β = −236.94, 95% HPD interval = [−620.82, 21.64], pMCMC = 0.134; Figure 3), meaning juveniles were no more likely to masturbate than adults (of the 108 records with identified age classes, all juvenile records involved masturbation compared to 97% of adult records).

A primary theory of the evolution of masturbation in organisms is that it correlates with a high sex drive, that can be associated with breeding system. We found that species that exhibit social monogamy, the most common mating system in birds, (Brouwer and Griffith 2019; Griffith et al. 2002) are less likely to masturbate (β = −203.16, 95% HPD interval = [−436.21, −0.15], pMCMC = 0.030; Figure 3), with species with multiples mates being more likely to masturbate (β = 162.12, 95% HPD interval = [−7.53,375.58], pMCMC = 0.050; Figure 3). This is reinforced by the finding that species with long‐term pair bonds were also less likely to masturbate (β = −214.96, 95% HPD interval = [−426.20, −21.97], pMCMC = 0.006; Figure 3).

When testing the effect of captivity on likelihood of masturbation across birds, we find that wild individuals are more likely to masturbate than captive (β = 277.94, 95% HPD interval = [40.82, 490.87], pMCMC < 0.001; Figure 3). Further, we find a tendency for hand‐reared birds to be less likely to masturbate than their parent‐reared counterparts (β = −269.69, 95% HPD interval = [−590.76, 0.78], pMCMC = 0.056; Figure 3), which, despite the relatively weak association, suggests that the behaviour is unlikely to be an artefact of unnatural captive conditions.

Finally, when examining the effect of social condition or environment on likelihood of masturbation across birds in captivity, we find no evidence to suggest that the presence or absence of other individuals (regardless of sex or species) influences the probability of masturbating (all pMCMC > 0.1; Figure 3).

## 4 Discussion

Our study is the first of its kind to examine the prevalence of masturbation within birds and investigate the evolutionary occurrence and persistence of this behaviour across taxonomic orders within this class. Through the multifactorial approach of comprehensive reviews of both published literature and online accounts, coupled with targeted surveys, we find masturbation is a strongly phylogenetically conserved behaviour characterising major groups of birds, suggesting that it is a widespread and naturally occurring behaviour. This is supported through the findings that masturbation occurs in both males and females, though males are more likely to masturbate, and that there is a clear link between mating system and likelihood of masturbation within a species. We also find some evidence that masturbation is more often lost than gained over evolutionary time, but we note that this result could also be influenced by unobserved masturbation in some species. For instance, if all species of a clade masturbate but some have merely not been observed to do so, those species might appear to have lost the behaviour based on our dataset. Perhaps most crucially, our results highlight that masturbation is a common occurrence across avian taxa and is more likely to occur in wild individuals. Masturbation is therefore not a maladaptive response to captive living, and this finding can have profound implications for captive animal welfare.

### 4.1 The Prevalence and Persistence of Masturbation in Birds

We used several parameters within our dataset to test whether masturbation persists across avian taxa through increasing reproductive success in any manner. Mating systems within birds are fairly complex. Whilst social monogamy is the dominant mating system with estimates of occurrence in over 90% of avian species (Lack 1968), extra‐pair copulations has also been estimated to occur in 90% (Griffith et al. 2002), resulting in mixed parent offspring through extra‐pair paternities (Akçay and Roughgarden 2007). If masturbation occurred more frequently in socially monogamous species, it may suggest it serves as a function, for example, to reinforce pair bonds, with the potential for old sperm removal in males to increase the fertilisation success of subsequent matings with their partner. This would particularly be the case for those with long‐term pair bonds. However, we find that masturbation is more likely to occur in species with multiple mates. This finding lends some support to both the Sexual Outlet Hypothesis (that explains the proximate reason for masturbation, that is, why an individual would choose to masturbate) and the Postcopulatory Selection Hypothesis (that addresses the adaptive function of masturbation, that is, why masturbation evolved and has been retained across the course of evolution).

However, our dataset spans reports from both captive and wild birds, with individuals therefore being in a range of social environments. With theories such as the Sexual Outlet Hypothesis predicting that individuals would not masturbate when they had access to a potential mate, the social environment in which an individual exists can have profound implications when examining evolutionary persistence. We found that masturbation is widespread across individuals from varying social environments, from solitary individuals to those in environments with multiple males and/or females, and even when housed with different species. The potential for the Sexual Outlet Hypothesis to explain all masturbation in birds is troubled as individuals are just as likely to masturbate when alone than when housed with a conspecific partner of the opposite sex. One potential explanation for this is that masturbation forms part of courtship behaviour in birds ‐ evolutionary conserved but subsequently lost in certain clades. Alternatively, there may be interactions between mating system and housing that we were unable to investigate with our data, for instance if some species may ‘expect’ to mate with a large number of partners and others do not, they may be more or less satiated with a given number of available social partners.

The Postcopulatory Selection Hypothesis (Brindle 2022, 2018; Sommer et al. 2022) posits that masturbation serves an adaptive function, and is compatible with scenarios in which individuals masturbate in the presence of a mate. This umbrella hypothesis encompasses two factors to ultimately predict that masturbation aids fertilisation and therefore reproductive success: the Sexual Arousal Hypothesis and the Sperm Quality hypothesis (Brindle 2022, 2018; Sommer et al. 2022). As females of many bird species have sneak copulations with subordinate males (Spezie and Fusani 2023) and/or exhibit extra‐pair paternities for their offspring (Akçay and Roughgarden 2007), the potential for female masturbation to serve as a function to speed up subsequent ejaculation in a mating (the Sexual Arousal Hypothesis), would prove beneficial and have substantial fitness benefits through increased likelihood in siring offspring. This provides an interesting explanation for this behaviour in wild individuals, and indeed, we do find wild birds more likely to masturbate than captive. In captivity, with restricted or no access to a potential mate, masturbation would still incur a clear time and energy cost, rendering the potential to speed up subsequent ejaculations in a mating unnecessary. It could therefore be suggested that this evolutionary conserved behaviour is still retained when birds transition from wild to captive environments, as birds of many species in captivity are often only a very low number of generations removed from the wild, and therefore still retain wild behaviours.

The second factor of this umbrella hypothesis focusses on sperm quality. The Sperm Quality Hypothesis states that ejaculatory male masturbation pre‐copulation improves the quality of subsequent ejaculates through expelling inferior sperm. Whilst there is some evidence to support this theory in primates (e.g., humans, Baker and Bellis 1993; macaques, Thomsen and Soltis 2004; Dubuc et al. 2013), and although we find males indeed more likely to masturbate than females, we have few reports of birds masturbating to ejaculation, suggesting that the Sperm Quality Hypothesis is unlikely to be the only selective force driving the evolution of masturbation in birds.

It is important to note that of the theories that aim to explain the occurrence of masturbation in animals, the majority are exclusively focussed on explaining why masturbation occurs in males with little attention paid to females. Recently, there has been increased awareness of this issue (see Roth et al. 2023; Brindle et al. 2023), yet a dominance of one‐sided theorising of this behaviour persists. We observe that whilst males are indeed more likely to masturbate, this behaviour occurs readily in both sexes across taxonomic orders. Indeed, the majority of records from female birds indicated that they did masturbate, albeit not quite as high a proportion as in records of males (when no masturbation was recorded it was most often absent from both sexes), strongly suggesting the masturbation by female birds is not unusual. While research into primate masturbation highlighted the lack of reports on females in peer‐reviewed literature (Brindle et al. 2023), we observed an equal split in data representation across the sexes in birds. This demonstrates clearly that our findings cannot simply be a result of data bias. It is vital that more attention is paid to female sexual behaviour if we are to understand the evolutionary drivers of this behaviour at a broad comparative scale across the sexes.

### 4.2 Masturbation Is Not a Maladaptive Response to Captivity

A focal point of this study was to draw upon the expertise of bird hobbyist breeders and keepers to investigate instances of masturbation in captive individuals. As this behaviour is so widely documented in hobbyist forums, groups and through avian veterinary surgeons, a growing body of ‘folklore husbandry’ (Arbuckle 2013) practices have evolved over a sustained time period. These non‐evidence‐based practices are ingrained through time rather than knowledge and so can potentially contribute to sub‐optimal welfare of captive animals (Arbuckle 2013). Attitudes towards masturbation in captive birds, whereby masturbation is considered an abnormal behaviour that has developed in response to solitary captive living, is an example of folklore husbandry. As a result, birds are actively discouraged, punished and even given medical intervention to try to prevent this ‘behavioural problem’ (Jones 2020). One solution of providing a suitable mate is often suggested (Currumbin Valley Vet 2015), though invariably birds continue to masturbate, consistent with our results showing lack of effect of social conditions in captivity. Routine practices of discouragement initially begin with behavioural interventions, such as through removing toys, perches, limiting interactions or altering photoperiod (Abou‐Zahr 2022; Wilson 2001). Changing their diet is frequently suggested, as a high energy diet is anecdotally linked to masturbatory behaviour (Rosen 2012). The final suggestions are the most severe and require medical intervention through psychoactive drugs or hormonal therapy to suppress masturbation (Seibert 2007) and most extreme, de‐sexing (Currumbin Valley Vet 2015).

Our dataset contains the greatest number of instances of masturbation from parrots, which are widely considered to be sexually stimulated through the scratching or stroking of areas around the rump that is thought to initiate masturbation in many individuals (Hoppes 2021). Whilst it could be argued that these behaviours can imitate mutual or allopreening, courtship and pair‐bonding behaviours that birds would exhibit with a partner, these can be replicated in birds bonded to their owner (this could particularly be the case for human hand‐reared individuals) (Hoppes 2021). We find that masturbation is indeed a natural behaviour that is widespread across avian taxa, occurring in both captive and wild birds, and is therefore not a maladaptive response to captive living. Therefore, the use of these potentially invasive mechanisms to stop captive birds displaying a natural behaviour have profound welfare implications, since high welfare typically requires the ability to perform natural behaviours where these are not detrimental (Wensley 2008).

Our study is the first of its kind to explore the widespread occurrence and evolutionary persistence of masturbation behaviour in birds across taxonomic orders. Our findings indicate that the proximate mechanism of masturbation may be to serve as a sexual outlet in response to a high sex drive. We also find support for evolutionary hypotheses, with masturbation appearing to be a flexible behaviour (within and between species) with different adaptive functions depending on life history attributes. In particular, masturbation appears to be associated with increased postcopulatory selection pressure. Most poignantly, we illustrate that masturbation is an evolutionarily conserved, natural trait present across the avian class and is not simply a pathological behaviour occurring in individuals experiencing ill health in response to a captive environment.

## Author Contributions

**Chloe Heys:** conceptualization (lead), data curation (lead), formal analysis (supporting), investigation (lead), methodology (lead), project administration (lead), resources (equal), supervision (equal), validation (equal), visualization (equal), writing – original draft (lead), writing – review and editing (lead). **Kevin Arbuckle:** conceptualization (equal), data curation (supporting), formal analysis (lead), investigation (equal), methodology (equal), project administration (equal), resources (equal), software (lead), supervision (equal), validation (equal), visualization (equal), writing – original draft (supporting), writing – review and editing (supporting). **Matilda Brindle:** formal analysis (supporting), investigation (supporting), writing – original draft (supporting), writing – review and editing (supporting). **Tom A. R. Price:** conceptualization (equal), data curation (supporting), investigation (equal), methodology (equal), project administration (equal), resources (equal), supervision (lead), validation (equal), visualization (equal).

## Conflicts of Interest

The authors declare no conflicts of interest.

## Supporting information


**Data S1:** Questionnaire disseminated to survey avian experts to quantify how widespread masturbation is across avian taxa. This questionnaire was disseminated to all avian experts including academics, practitioners and to zookeepers via the BIAZA (British and Irish Association of Zoos and Aquariums) network.

**Data S2:** Bayesian stochastic modelling outputs reporting the variable, parameter, posterior mean, lower 95% CI, upper 95% CI and the *p* value.

**Data S3:** Summary of sample sizes of each species included in the analysis, along with corresponding relevant information. This includes species name, presence/absence of masturbation (Y/N), sex (M/F), number observed, wild/captive origin, group size, housing condition (housed with opposite/same sex individuals), rearing origin (hand/parent reared), age (adult/juvenile) and the source (either expert, citizen science or literature).

## Data Availability

All data used has been submitted to Figshare data repository: https://doi.org/10.6084/m9.figshare.30569984.
