# Supplementary material for: The Evolution of Masturbation in Birds
Source: Ecol Evol. 2026 May 31;16(6):e73693. doi: 10.1002/ece3.73693 (PMC13240144; doi:10.1002/ece3.73693)
Supplement: Supplementary file 1 — Data S1: Questionnaire disseminated to survey avian experts to quantify how widespread masturbation is across avian taxa. This questionnaire was disseminated to all avian experts including academics, practitioners and to zookeepers via the BIAZA (British and Irish Association of Zoos and Aquariums) network. [file ECE3-16-e73693-s002.docx]

**Questionnaire about masturbation in birds**

**What we want to know:** Please tell us about any bird species you have seen masturbate, or any species where you are reasonably confident you would have observed masturbation if it occurred regularly in that species (due to very frequent observation of the species). If you are expert in multiple species, it would be great if you would tell us about as many of them as you can, using multiple sheets if you want. If any of the information requested is unknown, simply leave this box blank. If you work with a large collection, please respond with a list of species you have seen masturbating and/or which are confident (as above) do not masturbate, along with any of the information in the questionnaire you can readily provide.

**The behaviour we are looking for:** We define masturbation as a bird having sex with an inanimate object. Birds typically masturbate by rubbing their cloaca against an inanimate object, often a rock, branch, or something in their cage. This may lead to ejaculation in males.

| Species of bird: |  |  |  |
| --- | --- | --- | --- |
| Have you observed masturbation by a bird of this species (Y/N)? |  |  |  |
| If not, how confident are you that you would have seen it if it occurred (very/fairly/not very/not confident) |  |  |  |

**If you did see masturbation:**

| What was the sex of the birds that masturbated (M/F/both) |  |  |  |
| --- | --- | --- | --- |
| Approximately how many individuals of this species have you seen masturbate? |  |  |  |
| Were they in captivity (captive/wild)? |  |  |  |
| Were they solitary (alone/with same sex/with opposite sex/with both sexes)? |  |  |  |
| Were they hand reared (hand/parent)? |  |  |  |
| Were they adult (juvenile/adult)? |  |  |  |
| Do you consider the bird(s) to have been in good condition (good/bad)? |  |  |  |
| Are there any other details you think we should know? |  |  |  |

Please forward your completed questionnaire or any questions to:

Chloe Heys: c.heys@liverpool.ac.uk or Tom Price: t.price@liverpool.ac.uk
